# Heterocyclic molecules with ESIPT emission: synthetic approaches, molecular diversities, and application strategies

**DOI:** 10.55730/1300-0527.3585

**Published:** 2023-09-30

**Authors:** Nurettin MENGEŞ

**Affiliations:** 1 Science and Technology Research and Application Center (BİTAM), Necmettin Erbakan University, Konya, Turkiye; 2 Department of Biomedical Engineering, Faculty of Engineering, Necmettin Erbakan University, Konya, Turkiye

**Keywords:** ESIPT emission, synthesis, sensors, fluorescence

## Abstract

Excited-state intramolecular proton transfer (ESIPT) is one of the most essential emission processes in most circumstances because of its dual emission band in most cases and its high Stokes shifts. These distinguishing properties make ESIPT-based probes more suitable for a variety of applications, including analyte sensors, solid-state sensing mechanisms, optical technologies, and biomarkers for endogenous or exogenous compounds in various settings. As a result, researchers around the world are working on ESIPT emissions and developing different scaffolds for various applications or industry demands. This field of study is rapidly expanding and there is a need for an up-to-date review of synthesis methodologies and applications. This paper provides the highlights of ESIPT-based heterocyclic scaffolds, synthesis strategies, and application scenarios in the literature from 2017 to 2023.

## 1. Introduction

Fluorescence is a technique involving the popular luminescence family in which reliable molecules produce light from electrically excited states formed by a physical (for example, light absorption), mechanical (friction), or chemical mechanism. To create a fluorescence response, three traditional fluorescence processes have been used in the past: photoinduced electron transfer (PET), Foster resonance energy transfer (FRET), and internal charge transfer (ICT). There is also an additional mechanism that is newer than the others: excited-state intramolecular proton transfer (ESIPT) emission.

In the 1950s, Weller emphasized the significance of ESIPT involving salicylic acid [1]. Since then, ESIPT photophysics has been widely researched and used in a wide range of applications. For ESIPT emission to occur, the molecule must have particular properties. One of the most important factors is geometry, and the proton donor and acceptor groups of a molecule that may produce ESIPT must be in the correct shape. In this way, the acidic proton in the excited state of the molecule might migrate to the proton-acceptor group in a geometry close to itself. This mechanism consists of four phases. Following proton transfer in the excited state, the excited phototautomer emits photons in order to transform into its most stable isomer in the ground state, resulting in fluorescence emission. A pseudo-ring is projected to emerge between the two groups when the proton transfer occurs. This pseudo-ring often contains 5 or 6 rings. In compounds that create ESIPT, the donor groups are often OH or NH_2_, whereas the acceptor groups can be imine, carbonyl groups, and a nitrogen atom in the ring.

**Figure 1:**
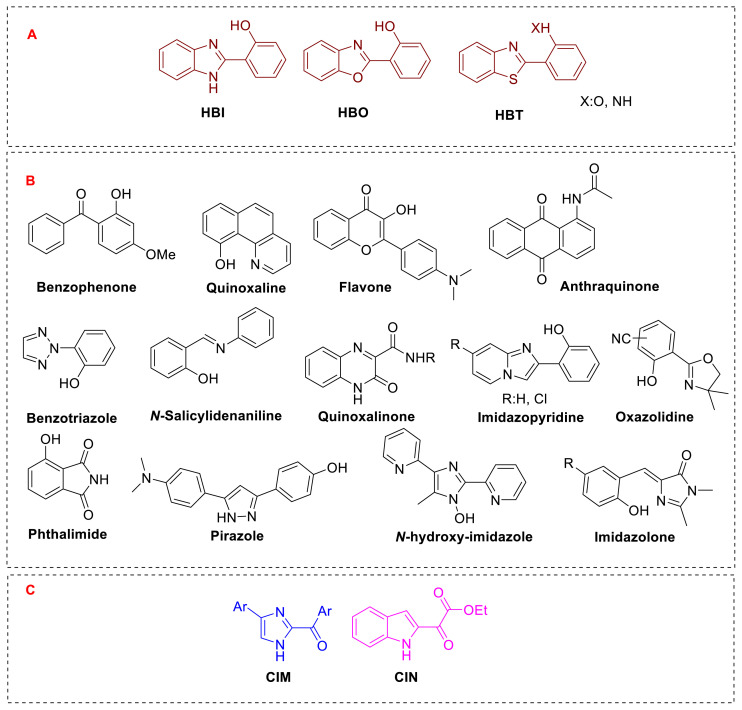
**A)** The most popular heterocycles for ESIPT emission; **B)** different substituents with different heterocycles for ESIPT emission; **C)** CIM and CIN structures for ESIPT emission.

If the excited molecule emits before ESIPT, it is referred to as enol emission, also known as normal emission. A new emission happens after ESIPT during the transition of the excited-state keto tautomer to the ground state. This emission is also known as keto emission. Following this emission, the ground-state keto form returns to the original enol form in the more stable ground state. This is known as reverse proton transfer (RPT). During this procedure, two distinct emissions are conceivable, and the energy of the keto emission will be smaller. Taking this process into consideration, novel compounds with various ESIPT structures and uses have been discovered in the literature (Figure 1).

ESIPT emission is considerable due to a number of causes, which may be listed as follows:

1. A few derivatives may have two different emission bands. The first emission is normal and follows Kasha’s criterion, but the second is ESIPT emission at a longer wavelength.
2. A chemical reaction may happen in the excited state via intramolecular proton transfer.
3. The most stable tautomer in the ground state may become the most unstable tautomer in the excited state.
4. In comparison to other fluorescence-producing compounds not having ESIPT emission, the Stokes shift value is relatively high, and this is a critical characteristic in optical applications.

In terms of ESIPT research, the most common heterocyclic compounds that have been developed are 2-(2-hydroxyphenyl)benzimidazole (HBI), 2-(2-hydroxyphenyl)benzoxazole (HBO), and 2-(2-hydroxyphenyl)benzothiazole (HBT), as well as their many derivatives. Besides these compounds, quinoline [2], benzophenone [3], flavone [4], anthraquinone [5], benzotriazole [6], quinoxaline [7,8], salicylidenaniline [9], pyrazole [10], and imidazolone [11] have been found to have ESIPT emission. Menges and his colleagues, on the other hand, recently found that, in addition to the ring systems indicated above, imidazole with a carbonyl in the C-2 position (CIM: C-2 carbonyl imidazole; CIN: C-2 carbonyl indole) and an indole ring with an oxalate functionality also emit ESIPT [12,13].

**Scheme 1:**
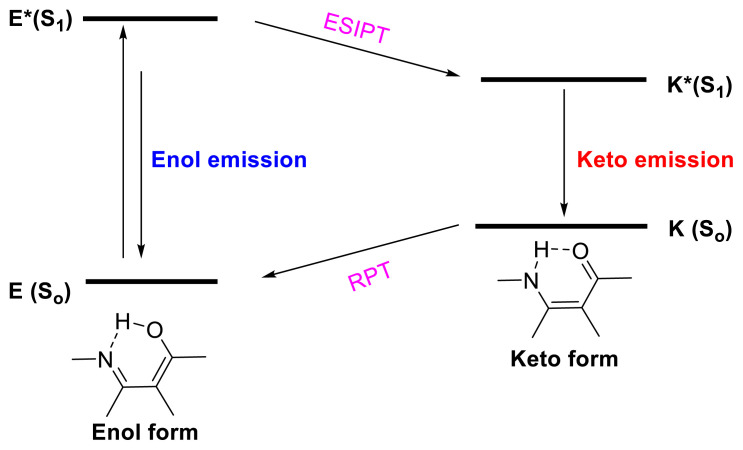
Proposed ESIPT mechanism.

Schemes 1A–1C present the most relevant examples of heterocyclic molecules possessing ESIPT emission. The most prevalent rings presented in the literature that emit ESIPT are the derivatives represented as HBI, HBO, and HBT (Scheme 1A). Scheme 1B, on the other hand, lists ESIPT-based compounds reported in the literature as part of a single study or many research projects. Apart from the compounds in Scheme 1, a boryl-substituted dithienylpyrrole skeleton has been reported to emit ESIPT [14]. There is a broad resemblance among the heterocyclic compounds that produce ESIPT in the literature (Schemes 1A and 1B). This likeness implies that the acidic proton attached to the ring’s heteroatom is transferred to the heteroatom of the other ring or to the ring’s carbonyl group. Finally, unlike the previous instances, molecules with ESIPT emission induced by the transfer of the acidic proton on the heteroatom of the heterocycle to a carbonyl group connected to the same ring and capable of free rotation are also presented in Scheme 1C. Among these compounds, indole was suggested for the first time as a possible source of ESIPT emission.

ESIPT emission has been the topic of many scientific investigations throughout the years, as well as numerous application studies. Taking into account the sample rings provided by the ESIPT emission, the synthesis of novel compounds with diverse heterocycles and functional groups is a focus of study today. There are other review studies on ESIPT-emitting compounds and their uses in the literature. The goal of the present review is to present a comprehensive and up-to-date study of ESIPT by addressing how numerous ESIPT-based compounds were synthesized, as well as their diverse fluorescence applications, between 2017 and 2023.

We chose papers published during the period specified and divided them into groups based on their fields of applicability. Ion detection, the creation of novel ESIPT-based scaffolds and the derivatization of existing ones, the detection of endogenous compounds, and other general applications were all taken into account in this manner. The applications and candidate molecules used in the case studies were not identical to one another.

## 2. Ion detection applications

**Scheme 2:**
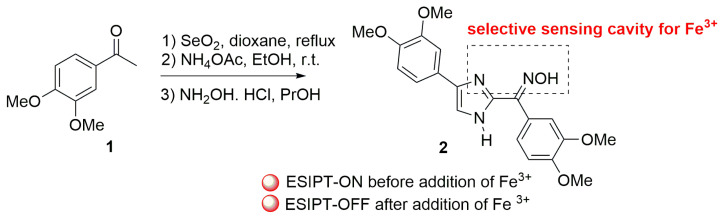
Imidazole-based probe for selective recognition for Fe^3+^.

Menges and his colleagues created a probe (molecule **2**) that can emit light via ESIPT emission in 2017. Unlike previous efforts, this ligand was reported to emit ESIPT owing to proton transfer between the NH and the oxime group of the imidazole ring at the C-2 position. The use of this ligand in the detection of various metals has been investigated and it has been published as a marker with the quenching of ESIPT, demonstrating selective action against ferric ions (Fe^+3^) among several other metals. Fe^2+^ and Fe^3+^ ions were chosen as metals, and selectivity to solely Fe^+3^ ions was also established (Scheme 2) [15].

**Scheme 3:**
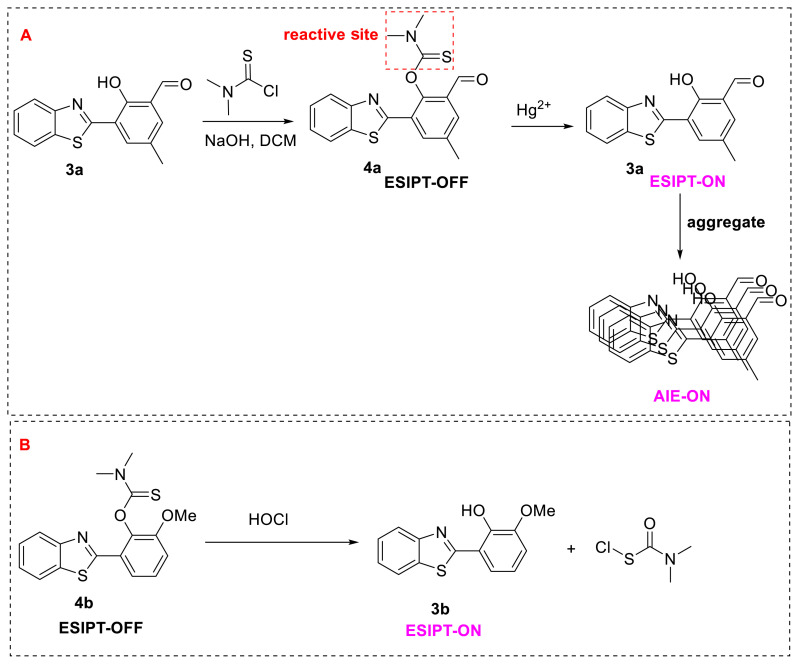
**A)** ESIPT-ON mechanism for Hg^2+^ and **B)** HOCl detection via a benzothiazole derivative.

Li, Tang, and their colleagues reported benzothiazole derivative probe **4a** based on the aggregation-induced emission (AIE) + ESIPT method for the detection of Hg^2+^. This probe is a chemosensor with a dimethylcarbamothioate group that reacts with Hg^2+^. By adding Hg^2+^ ions to the probe, the reactive group can be released, resulting in an ESIPT-emitted molecule. This molecule also formed an aggregation, which resulted in AIE emission. The probe’s selectivity demonstrated that it had strong antiinterference properties. Additionally, the limit of detection was predicted to be 2.85 × 10^−9^ M, with a Stokes shift of 170 nm. NMR and high-resolution mass spectroscopy (HRMS) were used to validate the release mechanism with the addition of Hg^2+^ to the probe solution. (Scheme 3A) [16]. A suitable probe for mercury detection, **4a**, was developed by replacing the methoxy group on the benzene ring in compound **4b** with an aldehyde group.

A different derivative with the same skeleton and the same reactive site (compound **4b**) was synthesized by James and his group for the detection of hypochlorous acid [17] (Scheme 3B). The authors developed luminous benzothiazole-based probe **4b** based on ESIPT for detecting HClO/ClO. The probe was found to have sensitivity and selectivity towards HClO/ClO, reacting entirely in 10 s and having a limit of detection (LOD) of 0.16 mM in HeLa cells. The probe detected both endogenous and exogenous HClO/ClO. Furthermore, the probe was also exhibited as a dual-input logic gate with Hg^2+^ and H_2_O_2_ inputs. Interestingly, Hg^2+^ alone produced a slow fluorescence response, but it required 430 min to get a substantial fluorescence response.

**Scheme 4:**
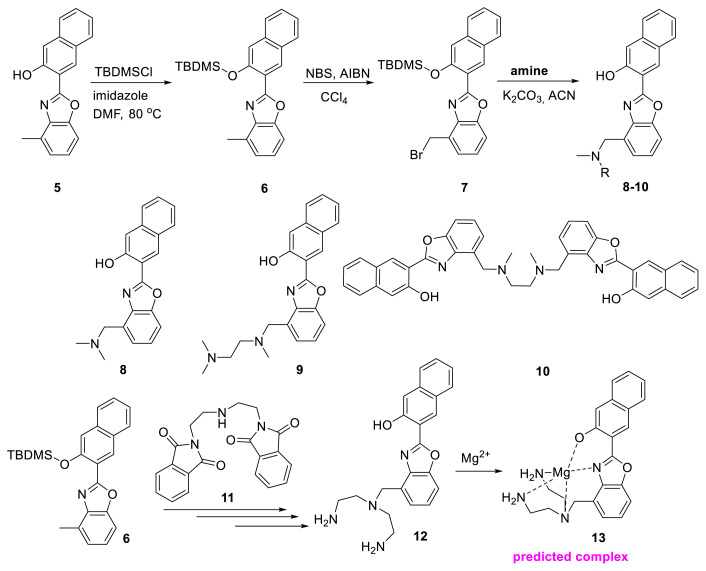
Detection of Mg^2+^ ions with benzimidazole derivatives having amine cavities.

Fusi and colleagues created 2-(2-hydroxy-3-naphthyl)-4-methylbenzoxazole derivatives with various amine moieties such as N,N-dimethylamine, N,N′,N′-trimethylethylendiamine, N,N′-dimethylethylendiamine, and N,N-bis(2-aminoethyl)amine. For the synthesis of probes **8–10** and **12**, these authors designed two different reaction paths. Finally, four different probes were synthesized (Scheme 4). In ACN and DMSO, the ligand emitted no light. The probe, however, demonstrated a strong fluorescence response with the addition of Mg^2+^ ions. According to the authors, Mg^2+^ may bind to the cavity of the diamine unit and the nitrogen atom of the oxazole ring, as well as abstract the hydrogen atom of the 2-naphthol unit (see predicted complex **13** in Scheme 4). For this reason, the ESIPT mechanism could not be developed; however, the authors did not go into depth on this issue. The probes may respond to Zn^2+^, Cd^2+^, and Mg^2+^ with varying intensities and emission bands. The authors also demonstrated the probe’s applicability on a paper-based fluorescent marker (Scheme 4) [18,19].

**Scheme 5:**
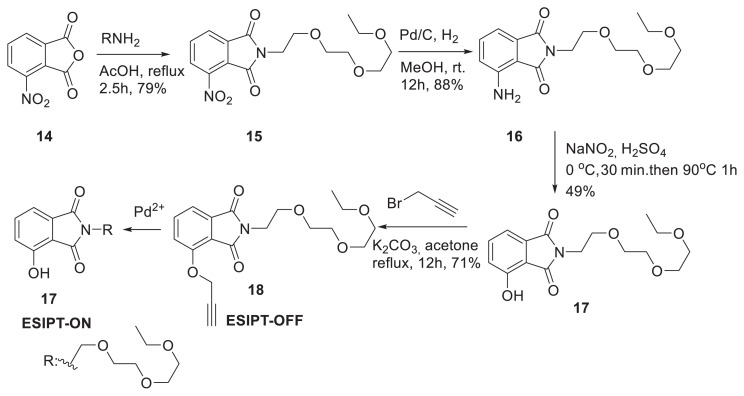
Design of a chemosensor with a propargyl group for detection of palladium ions.

Hand and colleagues created chemosensor **18** with an isoindoline-1,3-dione core and a propargyloxy unit for palladium ion ratiometric detection, which was synthesized through four different steps starting from compound **14**. Since propargyl units are only cleavable with palladium, the probe displayed great selectivity and sensitivity towards palladium ions [20]. Probe **18** contains ethylene glycol units for water solubility and has been effectively used for Pd^2+^ fluorescence imaging in live cells (Scheme 5) [21].

**Scheme 6:**
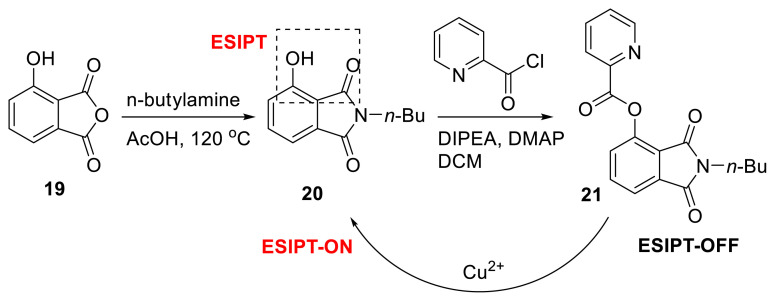
Sensing of Cu^2+^ ions with ESIPT-based phthalimide probe **21**.

Wang and colleagues studied a phthalimide derivative for its ESIPT-based metal recognition capacity. As a result, they created 2-butyl-1,3-dioxoisoindolin-4-yl picolinate (**21**) using only two easy procedures. The fluorophore is a hydroxy-phthalimide unit, while the recognition site for Cu^2+^ is a picolinate ester. The probe’s selectivity was strong and its detection limit was determined to be 31 nM. Furthermore, due to the probe’s chosen functionality, it demonstrated strong antiinterference capacity. Cu^2+^ detection was applied in a real water sample since the probe’s solubility in water was acceptable. The probe is a chemosensor because when it came into contact with Cu^2+^, picolinic acid was released from the probe, resulting in the ESIPT-ON structure (Scheme 6) [22].

**Scheme 7:**
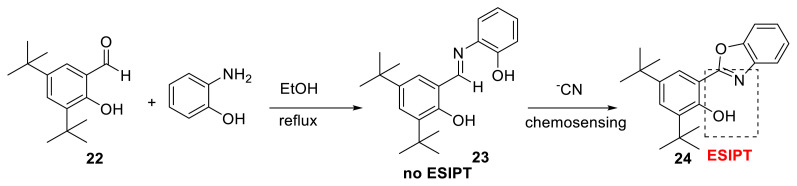
Imine-based chemosensor for detection of CN ions.

For ^−^CN detection, Schiff base optical probe **23** was constructed as a chemosensor. A surfactant-assisted method was used for detection in water. These authors observed that sensing for ^−^CN was time-dependent due to the cyclization process. They showed that the probe may work as a real-time colorimetric chemosensor and then as a fluorescent light-up chemosensor for CN generated by cyclization followed by ESIPT and aggregation-induced emission enhancement (AIEE) (Scheme 7) [23,24].

## 3. Molecular diversity and enhancement of ESIPT emissions

**Scheme 8:**
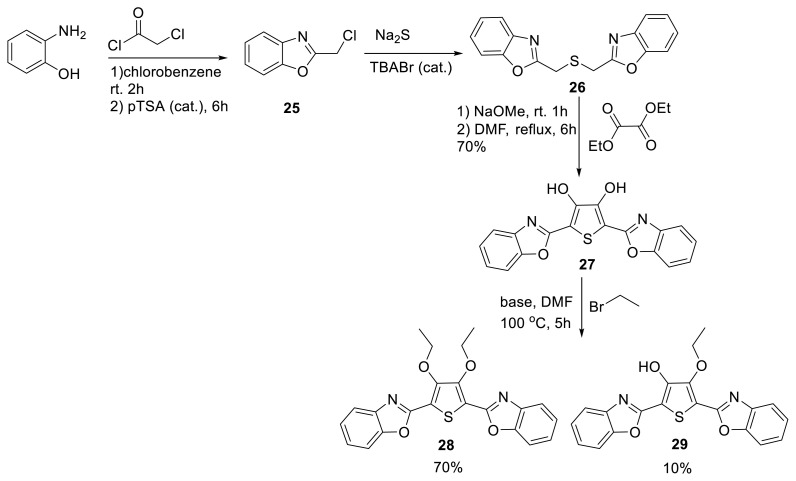
Synthesis of symmetric bisbenzoxazole-thiophene-diol and its derivatives for double ESIPT emission.

Chen and Hao synthesized compounds **27–29** for single and double proton transfer phenomena. They studied the molecules using fluorescence spectroscopy and lifetime measurements [25]. After that research, Jacquemin and his colleagues took into account the same skeleton for theoretical calculations in detail. The authors said that because this molecule had two ESIPT sites, the emission spectrum was complex, resulting in three nonequivalent tautomers. The authors described how the molecule had an initial double enol form and a single ESIPT enol-keto tautomer. The research encompassed extensive time-dependent density functional theory (TD-DFT) and post-Hartree–Fock approaches [ADC(2) and CC2] as well as structure–property examination employing various donor and acceptor groups (Scheme 8) [26].

**Scheme 9:**
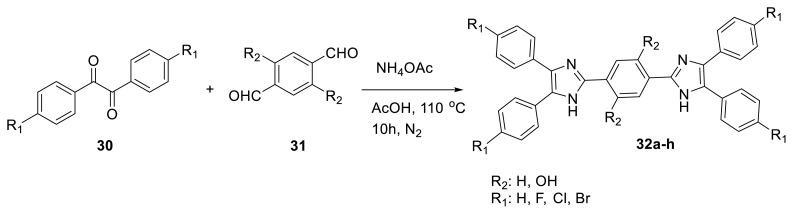
Synthesis of ESIPT-based imidazole derivatives with dual emission.

Pan and colleagues created imidazole derivatives **32a**–**32h** with dual ESIPT sites. The authors observed that dual ESIPT sites displayed full-color panel emissions from blue, green, and yellow to red, as well as white light in various solvents as controlled by enol-keto to keto tautomer emissions. The phototautomers’ lifetimes were measured at 0.8, 20, and 1900 ps. Theoretical simulations revealed a small energy barrier, corroborating the experimental findings. The dual ESIPT sensor’s applicability revealed selective detection of copper ions and solid-state fluorescence changes in response to organic vapor stimulation (Scheme 9) [27].

**Scheme 10:**
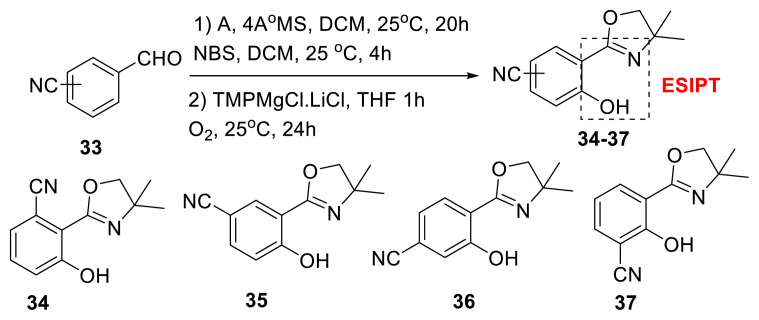
Synthesis of nitrile-substituted 2-(oxazolinyl)-phenols **34**–**37**.

Stauch, Nachtsheim, and colleagues discovered nitrile-substituted 2-(oxazolinyl)-phenols **34–37** to be ESIPT-based fluorophores with high solid-state emitters. The presence of CN functionality on the benzene ring in four different places results in four distinct regioisomers, **34–37**. As a result, the authors produced four isomers and tested their absorbance and fluorescence spectroscopy. Spectroscopic investigations revealed that two isomers (**34** and **35**) exhibited AIEE, one isomer (**36**) exhibited dual-state emitters (DSEs), and another isomer (**37**) exhibited aggregation-caused quenching (ACQ) fluorophores. In addition, the authors revealed that **32** possesses a minimalistic ESIPT-based fluorophore with a high quantum yield in the solid state (87.3% at λ_em_ = 491 nm). The Stokes shift of **34** in solution was determined as 160 nm. Temperature-dependent emission mapping, TD-DFT calculations, and crystal structure analysis were provided for further information on emission mechanisms. TD-DFT computations revealed that ESIPT emission resulted from the S1 state of the keto tautomer. A single crystal structure investigation revealed that the molecule has a well-formed molecular structure and an exceptionally well-organized crystal lattice (Scheme 10) [28].

**Scheme 11:**
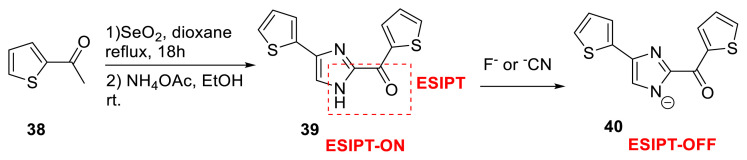
Imidazole derivative for ESIPT emission and its detection mode for anions.

Menges and his colleagues created a novel heterocyclic skeleton for an ESIPT emission. They used a one-pot, two-step technique to create imidazole derivative **39** for ESIPT-based probes. The probe emits light using the ESIPT process, which employs an NH proton as a proton donor and a C-2 bonded free-rotatable carbonyl group as a proton acceptor. Its skeleton was revealed in the literature for the first time since it utilized distinct functional groups that had not before been acknowledged. The authors demonstrated that the substitution of methyl for the proton donor group and the reduction of the proton acceptor carbonyl group damaged the ESIPT emission. Those derivatives did not emit light in the visible region. Independent experiments and TD-DFT calculations revealed that the planned skeleton emits light via an ESIPT process with a barrierless energy change, resulting in a single emission band (Scheme 11) [29]. The probe’s applicability for distinguishing several anions was detailed by the authors. Spectroscopic tests revealed that probe **39** is susceptible to basic anions such as fluoride and cyanide. The selectivity of the aforementioned anions over other possible anions was discovered, and a sensing mechanism was given using ^1^H NMR and TD-DFT calculations (Scheme 11) [13].

**Scheme 12:**
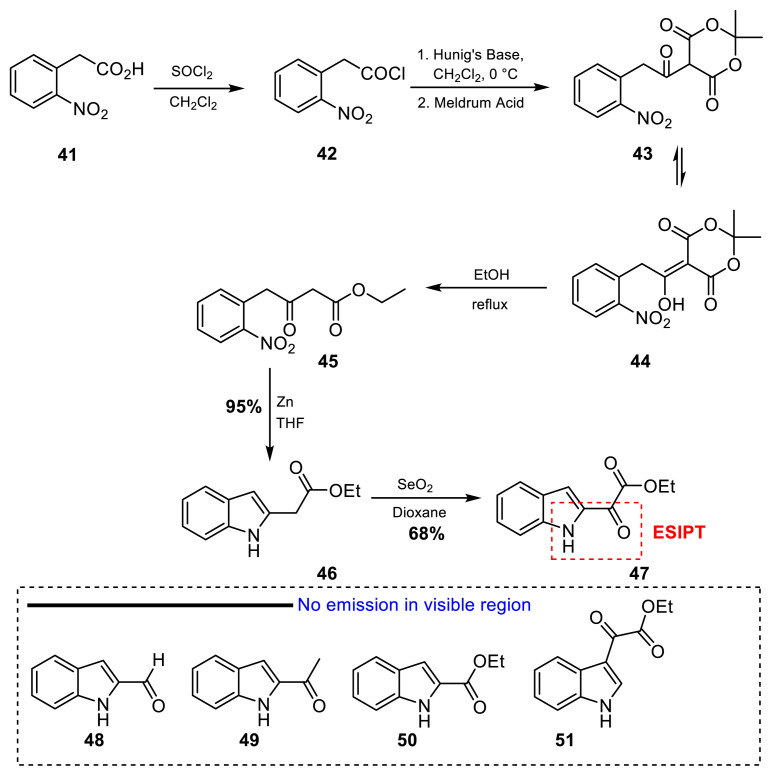
Investigation of indole derivatives for possible ESIPT emission.

Menges and his colleagues looked for a novel heterocyclic core with ESIPT emission. Thus far, numerous different heterocyclic skeletons have been employed in the literature. However, there was no indole derivative relevant to ESIPT emission at the time. They created indole derivatives with carbonyl inserted at the C-2 position. The presence of formyl (**48**), acetyl (**49**), and ester (**50**) groups at the C-2 position of the NH-indole derivatives did not result in emission in the visible region. The authors expanded the conjugation by employing an ethyl-oxalate group at the C-2 position (compound **47**). Unexpectedly, they discovered that this molecule emits light in the visible region. Many independent experiments were used to explore the emission process, one of which was shifting the location of the oxalate group, which resulted in no emission (compound **51**). Furthermore, TD-DFT calculations confirmed that ESIPT emission can occur, having barrierless energy changing between the phototautomers. The first indole skeleton for ESIPT emission was reported and its spectrum had only one emission band in the visible region (Scheme 12) [12].

**Scheme 13:**
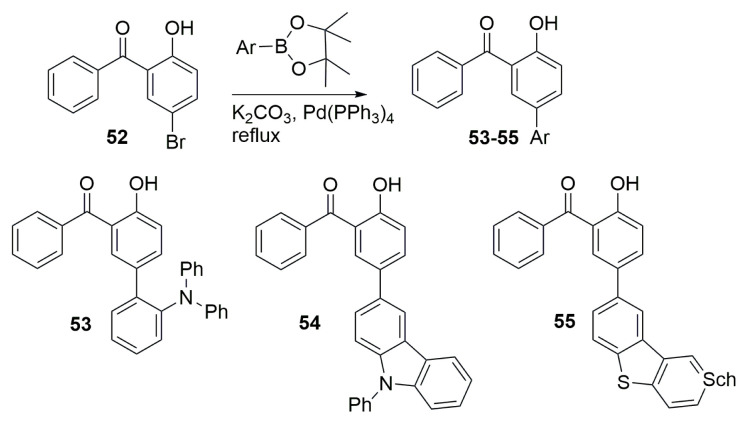
Benzophenone derivatives **53**–**55** for ESIPT emission.

Hydroxybenzophenone compounds have been studied for ESIPT emission. Huang and colleagues created numerous derivatives of the 2-hydroxybenzophenone skeleton (**53–55**) in order to investigate their structure–property relationships. The Suzuki–Miyaura reaction was used to create a hydroxy benzophenone core including triphenylamine, carbazole, and dibenzothiophene. Fluorescence spectroscopy of the derivatives in solution revealed dual-emission spectra, indicating the ESIPT process. When stimulated at low excitation energy, derivatives of the enol-type stabilized by intramolecular hydrogen bonds exhibited a wide emission spectrum in solid states. While hydroxy benzophenone with a triphenylamine moiety (**53**) showed excitation wavelength dependency, **54** and **55** did not, prompting the authors to speculate that inflexible and weaker donor characteristics may limit this process. The authors stated that having a triphenylamine core, which had greater donor strength than the others, caused an apparent color shift gradually from yellow to orange-red as the excitation power increased from 1 to 15 mW/cm^2^ (Scheme 13) [30].

**Scheme 14:**
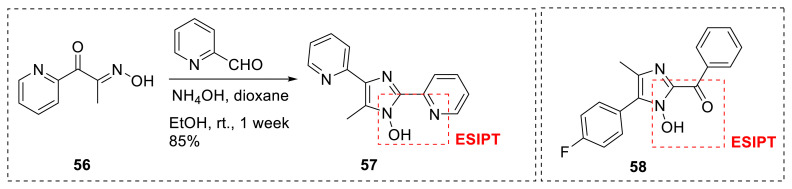
N-Hydroxy imidazole derivatives for ESIPT emission.

Shekhovtsov, Bushuev, and colleagues created tetra-substituted imidazole-N-hydroxy **57** for its ESIPT potential. The proposed skeleton includes a hydroxy group as a proton donor group and a pyridine ring at the C-2 position of an imidazole ring as a proton acceptor group. The authors stated that the proposed molecule can have two prototropic tautomers, namely N-hydroxy and N-oxide versions. According to the authors, the emission came from anti-Kasha S_2_ → S_o_ fluorescence in the N-oxide form as a result of a large S_2_ → S_1_ energy gap. The Stokes shift was discovered to be 60 nm, the shortest value among known ESIPT-based fluorophores (Scheme 13) [31]. The same research team discovered that adding a carbonyl group to the C-2 position of imidazole ring **58**, as stated by Menges and his research group for the identical heterocyclic core, resulted in ESIPT-based emission, as well. The designed molecule emitted light in solution and in solid state with a maximum wavelength range of 455–470 nm. The authors observed that the molecule in the MeCN solution displayed excitation wavelength-dependent emission with a shoulder of about 400 nm under high energy excitation (Scheme 14) [32].

**Scheme 15:**
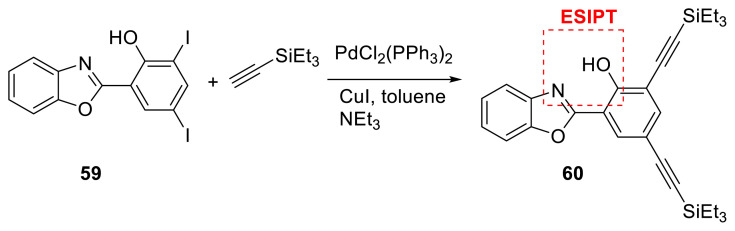
Benzoxazole derivative with alkyne units for ESIPT emission.

Ulrich and his colleagues created a molecular fluorophore (compound **59**) by combining benzoxazole with a 2-hydroxybenzene unit. The authors discovered that substituting triethylsilyl-ethynyl functionalities at positions 3 and 5 of the phenol moiety (compound **60**) increased the fluorescence in the solid state compared to unsubstituted derivatives. Another point to mention is that strong fluorescence emission was preserved in protic solvent such as ethanol and as 1% doped in a poly(methyl methacrylate) (PMMA) film. Researchers highlighted how random lasing studies of an ESIPT emitter indicated the presence of stimulated emission occurring beyond the pumping energy density threshold (ρth ≈ 300 μJ cm^−2^) in the PMMA matrix (Scheme 15) [33].

## 4. Detection of endogenous/exogenous molecules

**Scheme 16:**
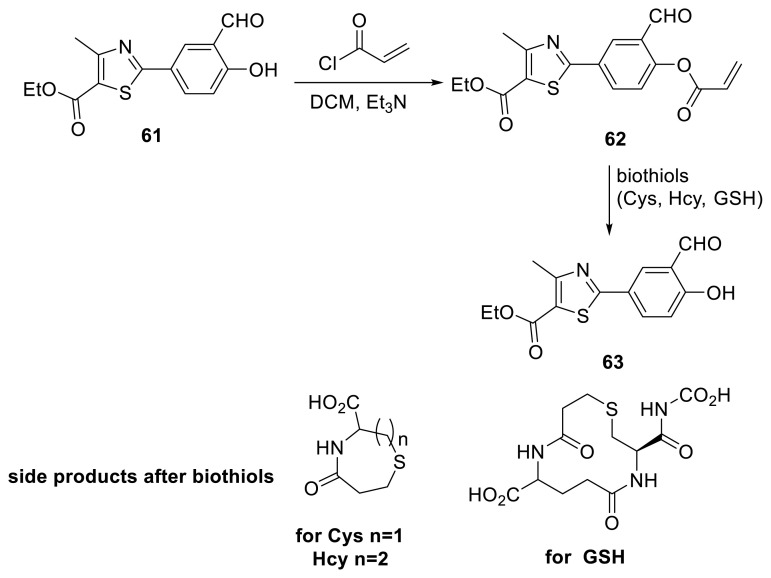
Detection of biothiols with acrylate unit through ESIPT emission (Cys: cysteine, Hcy: homocysteine, GSH: glutathione).

Hua, Na, and their colleagues reported employing ESIPT-based heterocyclic compound **62** to detect intracellular thiols in a selective and sensitive manner (Scheme 16) [34]. In that work, they synthesized several thiazole compounds that were used as a colorimetric and ratiometric fluorescent probe to detect biothiols in physiological fluids. The LODs for Cys, Hcy, and GSH were determined to be 0.156, 0.185, and 1.838 μM, respectively. The authors reported that NMR and MS investigations validated the ESIPT emission process.

**Scheme 17:**
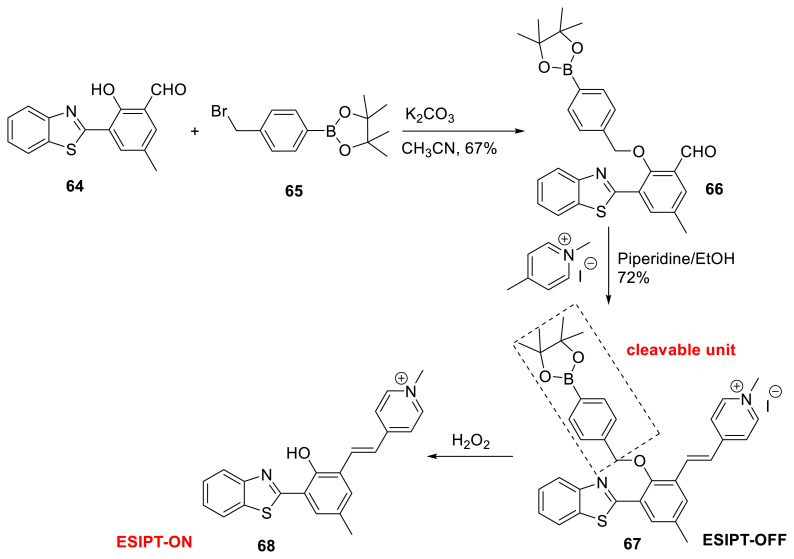
Synthesis of mitochondria-targeted chemosensor **67**.

Tang, Yan, and their colleagues investigated a mitochondria-targeted ratiometric fluorescence sensor including benzothiazole substructure **67**. The studied sensor has strong selectivity for H_2_O_2_ over other reactive oxygen/nitrogen species, anions, and biological thiols. The sensing mechanism was explored and it was claimed that the addition of H_2_O_2_ to the sensor’s solution initiated oxidative hydrolysis, followed by an elimination process that resulted in the release of ESIPT-featured fluorescent probe **68**. The sensor’s cell permeability was described, as was its use for exogenous and endogenous H_2_O_2_ in A549 cells (Scheme 17) [35].

**Scheme 18:**
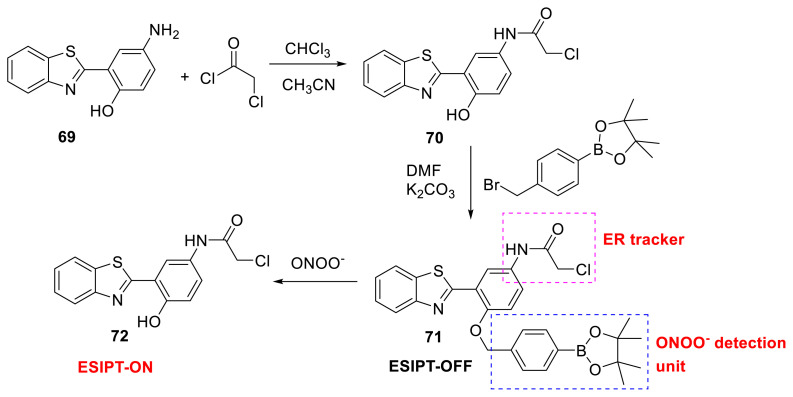
Synthesis of compound **71** for ER tracking and detection of ONOO^−^.

James and his colleagues created probe **71** to detect ONOO^−^ (peroxynitrite), a highly reactive nitrogen species that acts as a signaling molecule in a multitude of pathways throughout the body [36]. The probe has two vital functionalities, which are boronates and chloroacetamide. While boronates show good reactivity toward ONOO^−^ among HClO and H_2_O_2_, the chloroacetamide functional group is capable of a covalent attachment to biomacromolecules located at the endoplasmic reticulum (ER). Within seconds, the proposed sensor identified ONOO^−^ with a LOD of 21.4 μM, resulting in a ratiometric shift in fluorescence intensity. The addition of ONOO^−^ gave ESIPT-ON molecule **72**, which formed after releasing the boronate unit. The authors validated the sensor’s applicability in vitro by performing co-localization investigations with commercially available ER-Tracker Red utilizing HeLa cells. Lastly, they demonstrated 3D surface plot analysis, which revealed a significant overlap between the sensor and ER-Tracker Red (Scheme 18) [37].

**Scheme 19:**
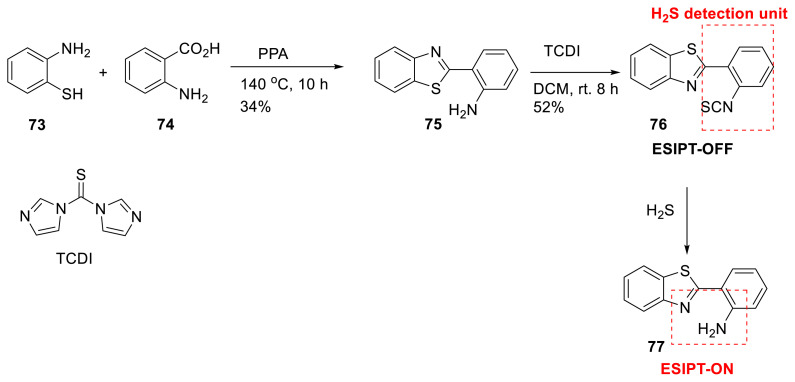
Synthesis of probe for detection of hydrogen sulfur in living media.

Jang and his colleagues created fluorescent probe **76** based on ESIPT emission for detecting hydrogen sulfide in a variety of working systems. The probe has a benzothiazole core with isothiocyanate as a detecting component. The probe has no fluorescence capabilities on its own, but the presence of H_2_S in the environment changes the isothiocyanate to the amine group, resulting in ESIPT emission, which serves as a marker for H_2_S. The sensing process was demonstrated using UV-Vis, fluorescence, and ^1^H NMR spectroscopic studies. The probe was built with a significant Stokes shift, a quick reaction time, and a low detection limit of 0–36 nM. The sensor’s applicability was tested in water samples and live cells (Scheme 19) [38].

**Scheme 20:**
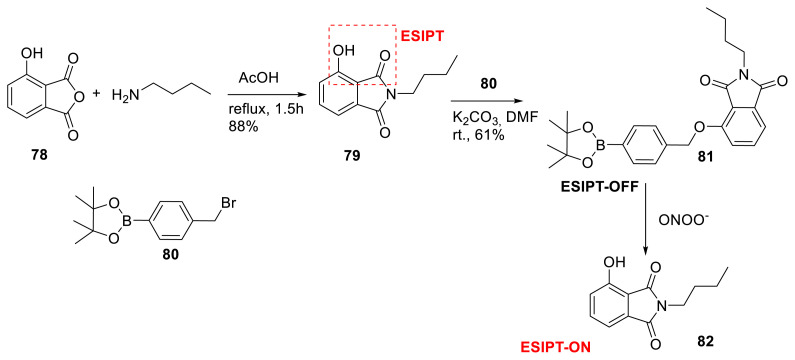
Synthesis of probe **81** for peroxynitrite detection.

Lim, Kim, James, and their collaborators demonstrated two-photon ESIPT-based fluorescent probe **81** for the detection of peroxynitrite, with a 4-hydroxyisoindoline-1,3-dione heterocyclic core. They explained that ESIPT-based fluorophores with two-photon excitation fluorescence (TPEF) are limited, and their goal was to discover a novel ESIPT-based probe with TPEF. They developed 4-hydroxyisoindoline-1,3-dione with a phenylboronic acid pinacol ester unit to detect ONOO^−^. The study revealed selectivity, sensitivity, and reaction time to ONOO^−^. In addition, the photostability of the probe under two-photon irradiation at 750 nm was demonstrated. The probe’s use was tested, and they discovered that it can image ONOO^−^ in HeLa cells and rat hippocampus slices at a depth of 110 μm (Scheme 20) [39].

**Scheme 21:**
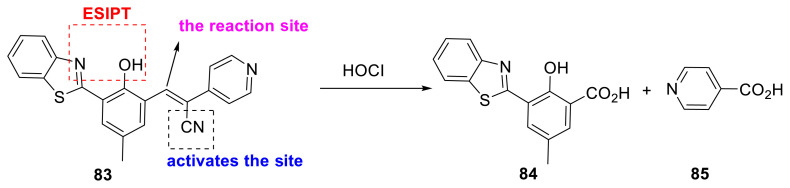
Benzothiazole derivatives for chemosensors for the selective detection of HOCl.

Yin and colleagues created a 2-(2’-hydroxyphenyl)benzothiazole molecule with a cleavable functionality (**83**) for detecting HOCl (hypochlorous acid). The CN group on the double-bond carbon atom in the cleavable unit renders the probe sensitive to the analyte. The probe’s emission was efficiently shifted to the red area, preventing interference from physiological autofluorescence. Probe **83** was evaluated in live cells for its applicability, and the authors reported that the probe responded to endogenous HOCl (Scheme 21) [40].

**Scheme 22:**
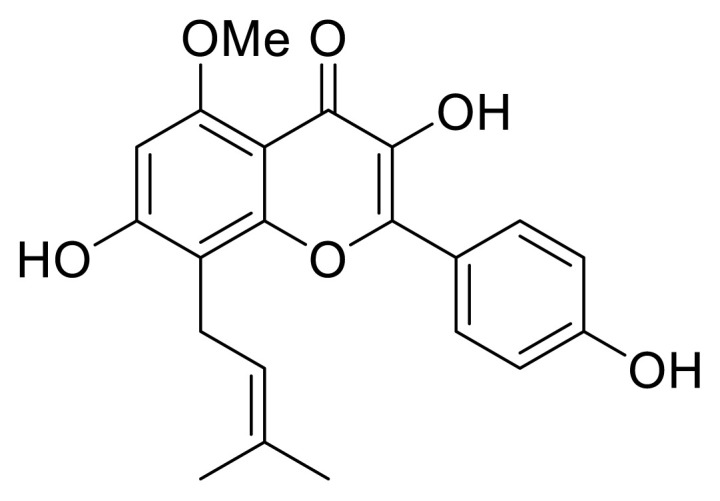
ESIPT-emitted flavonoid for detection of heptad-interfaced G-quadruplexes.

Shao and his colleagues reported ESIPT-based flavonoids for recognizing heptad-interfaced G-quadruplexes. Although these authors utilized various flavonoids for their sensing study, sophoflavescenol with a prenyl unit gave the best result. The absorbance of sophoflavescenol increased when it was added to the medium including G-quadruplexes. The authors claimed that prenyl and phenol units on the flavone core can be restricted without any rotation of single bonds when the sensor is integrated to G-quadruplexes. Integration and restriction of molecular rotation triggers ESIPT emission, resulting in the detection of the DNA quadruplexes (Scheme 22) [41]. The remaining flavonoids tested (10 derivatives) lacked a prenyl unit and showed no sensitivity to G-quadruplexes.

**Scheme 23:**
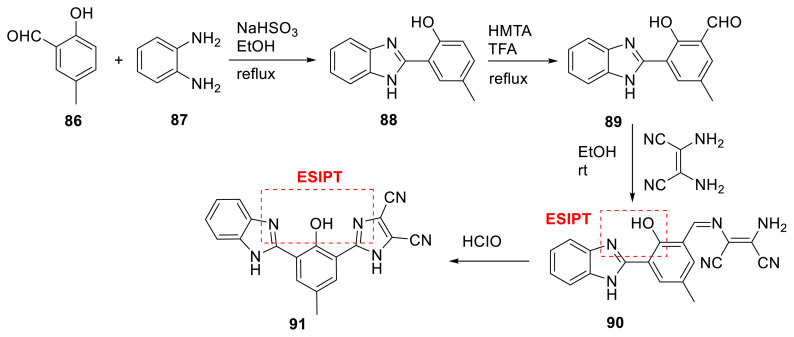
Benzimidazole derivatives for HOCl detection.

Gong and colleagues created novel benzimidazole-based fluorophore **90**. It has an ESIPT emission and a reactive site called diaminomaleonitrile. The molecule was created as a chemosensor for detecting HOCl. The reactive site of diaminomaleonitrile was reacted with HOCl, yielding a new imidazole ring in the medium and giving compound **91**. This reaction exhibited a strong fluorescence emission band, which assisted the analyzer in detecting HOCl. The authors used MS, HPLC, and ^1^H NMR spectroscopy to identify a unique sensing mechanism (Scheme 23) [42].

**Scheme 24:**
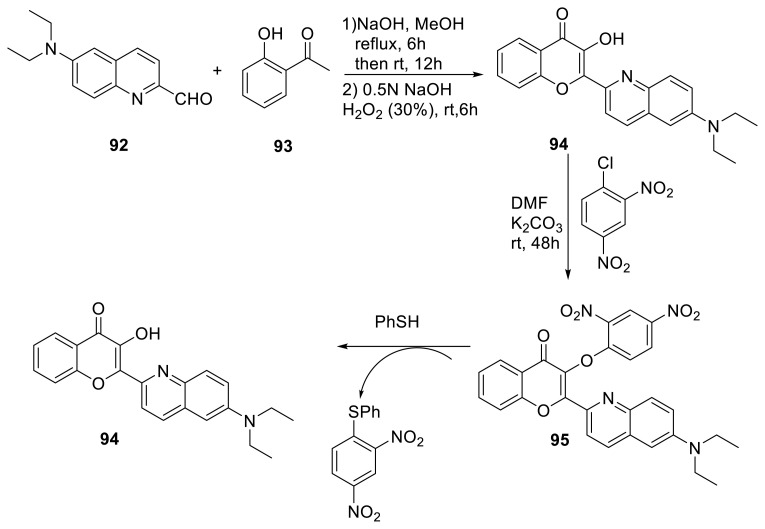
New ESIPT-based probe for thiol detection.

Two different research groups have studied the same skeleton. The first report was published by Liang and his research group. They synthesized a novel 2-(6-(diethylamino) quinoline-2-yl)-3-hydroxy-chromen-4-one (**94**) capable of emitting red to near-infrared fluorescence emission based on the ESIPT mechanism. The authors changed the hydroxy group using 2,4-dinitrophenol, and the resultant product **95** was used as a chemosensor for PhSH detection. The addition of PhSH to the probe solution liberated the dinitrobenzene moiety with thiophenoxy, resulting in the ESIPT-ON mechanism. The authors discussed the selectivity and sensitivity of the probe and found the LOD value as 7.2 nM (Scheme 24) [43]. Another research group also studied the same skeleton and they discussed the same reactivity for the probe. In addition, DFT and TD-DFT calculations were used to describe the sensing process. On the other hand, excited dynamic simulations revealed that the proton transfer process took 71.5 fs. The authors demonstrated that the proton transfer energy barrier is low, allowing ESIPT emission to occur quickly, resulting in only one emission peak being detected in the experiment, as supported by DFT simulations (Scheme 24) [44].

**Scheme 25:**
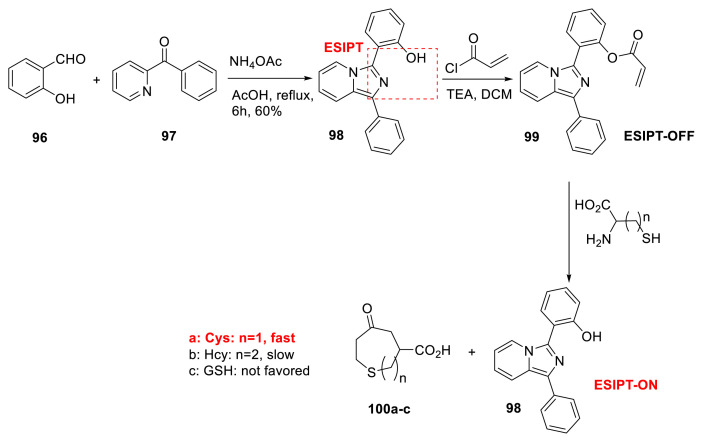
Selective detection of thiols with an imidazo[1,5-α]pyridine derivative as a chemosensor.

Yang and colleagues developed imidazo[1,5-α]pyridine skeleton **98** for ESIPT-based turn-on detection of cysteine among other thiols such as Hcy and glutathione. Molecule **99** was substituted with an acrylate unit, which could be attacked by cysteine and cause the produced moiety to be released from the heterocyclic ring (**100a**–**100c**). This reaction resulted in ESIPT emission, and cysteine was eventually identified because of its fastest reaction response compared to the other thiols (Scheme 25) [45]. Another research group reported that practical testing and theoretical calculations offered good evidence for the sensing mechanism (Scheme 25) [46]. A similar method was applied using a different ESIPT-based skeleton. In that investigation, a 2-hydroxy acetophenone derivative with an acrylate unit was used to recognize cysteine [47].

**Scheme 26:**
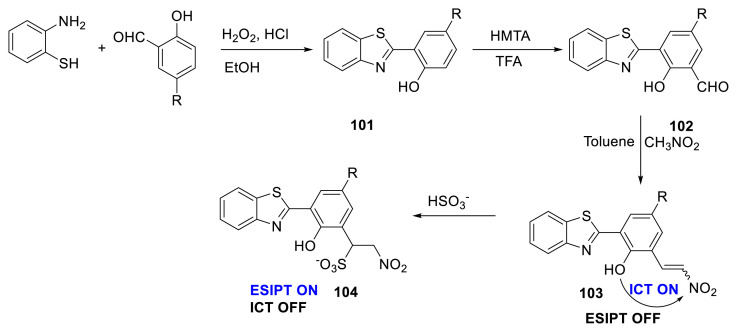
Design of a new probe for HSO_3_^−^ ions.

Novel fluorescent probe **103** for HSO_3_^−^ with HBT derivatives as the fluorophore and a nitroolefin moiety as the receptor was developed. The authors reported that the probe displayed exceptional features, including a substantial Stokes shift, a low detection limit, and strong selectivity and competition for HSO_3_^−^ compared to various heterogeneous anions and biothiols. The authors stated that the conjugate addition of HSO_3_^−^ to the probe’s nitroolefin caused the fluorescence amplification and UV-Vis spectral alteration. Furthermore, the preliminary biological investigations of that study demonstrated the use of the probe in biological systems by monitoring intracellular HSO_3_^−^ (Scheme 26) [48].

**Scheme 27:**
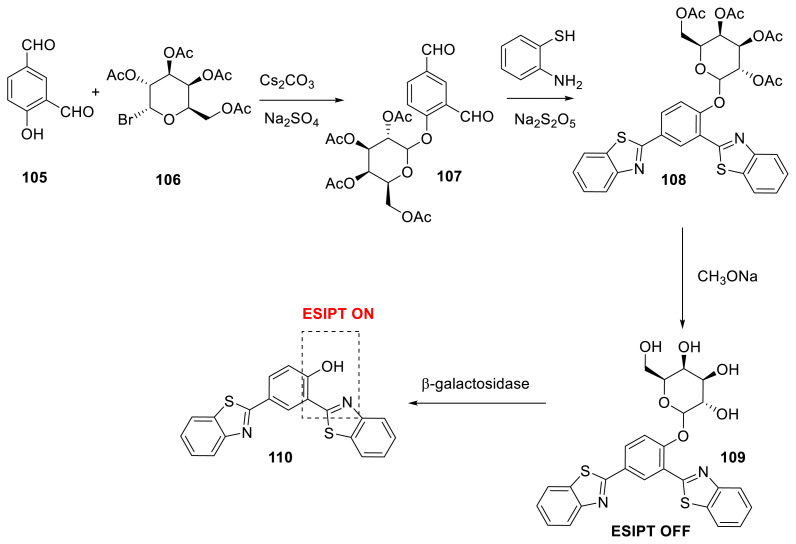
Detection of galactosidase enzyme with new ESIPT-based probe.

Novel fluorescent probe **109** based on the ESIPT mechanism and employing the HBT derivative as the signal reporter and D-galactose **106** as the response group was reported. Although the probe exhibited modest background fluorescence, it showed heightened fluorescence when the enzyme interacted with it. The authors also reported that the probe had a substantial Stokes shift (197 nm), a quick reaction speed, and strong selectivity. The authors proved that the probe could detect D-galactose in live cells and be employed in tissues in biological investigations (Scheme 27) [49].

## 5. Miscellaneous applications

**Scheme 28:**
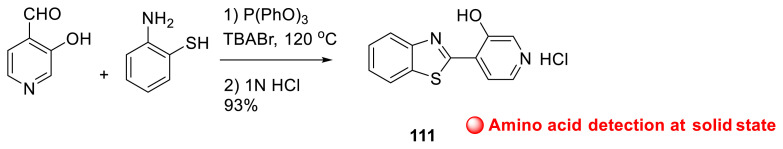
Detection of amino acids with a benzothiazole core with ESIPT emission.

Akutagawa and his colleagues synthesized 2-(2-hydroxypyridyl)benzothiazole molecule **111**. In solid state, this molecule does not exhibit any fluorescence characteristics. Nevertheless, the neutral state of the sensor can detect amino acids via solid-state grinding in an agate mortar, which creates hydrogen-bonding complexes at the pyridine site. UV exposure after grinding revealed variances among the amino acids, with differing pKa values of amino acids playing a crucial role. The authors also demonstrated a reversible H_2_O adsorption/desorption cycle in the H_2_O-adsorption isotherm at 298 K. In addition to the aforementioned investigations, a thin film of neutral form was used for detecting HCl vapor, and it was discovered that the sensor displayed bright green fluorescence when exposed to HCl vapor over a certain concentration (Scheme 28) [50].

**Scheme 29:**
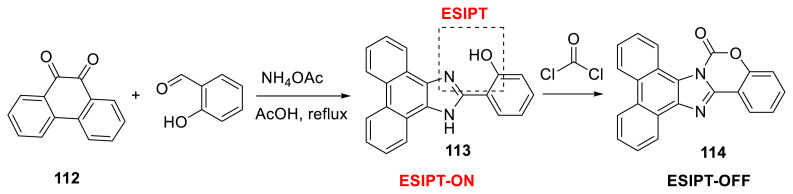
Synthesis of imidazole-based probe **114** for detection of phosgene.

Phosgene is an extremely poisonous gas that can cause serious health issues [51]. As a result, detecting it selectively is important. Li and colleagues developed imidazole-based fluorescent probe **113** capable of ESIPT emission for detecting phosgene in solution and gas phases via a single-step reaction strategy. The probe contains two key sites for recognizing the analyte. When phosgene was added to the probe’s solution, the emission changed to the blue area and detection was accomplished. The probe’s detection limit was 0.14 μM, and the sensing mechanism was confirmed using ^1^H NMR spectroscopy and HRMS. The authors created a fluorescent test strip to detect phosgene in the gas phase, which changes color from green to blue when exposed to handled-type UV radiation of 365 nm (Scheme 29) [52].

**Scheme 30:**
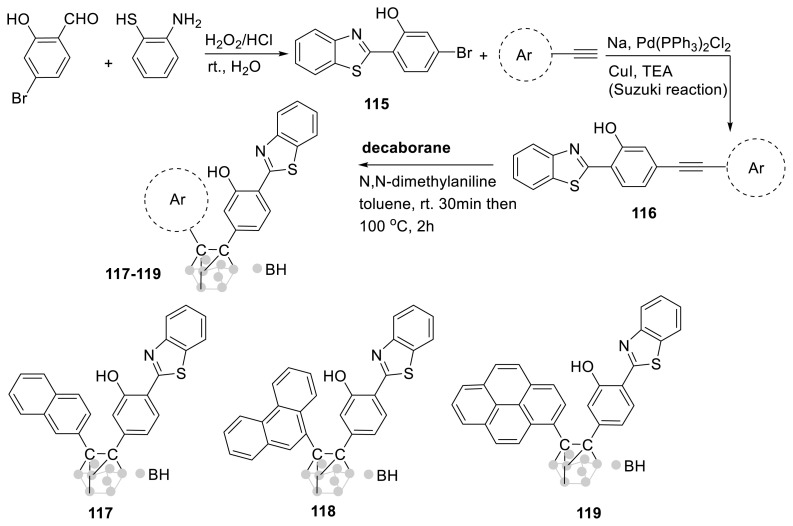
ESIPT-based probe having benzothiazole and carborane rings for recognition of mustard gas.

For the first time, hydroxy-benzothiazole derivatives [53] with an o-carborane ring were created for identifying mustard gas (2-chloroethyl ethyl sulfide). The probe’s electron acceptor is an ESIPT-based unit in **115**, and its electron donors are naphthyl in **117**, phenanthrene in **118**, and pyrene ring in **119**. The solid-state emission of three distinct ESIPT-based compounds was shown. All probes have porous structures and they differ in their solid-state emission characteristics. The probe detected mustard gas at a detection limit of 50 ppb and a reaction time of 5 s. The authors described a new technique for adjusting ESIPT by adding excited-state intramolecular charge transfer (ESICT) emission in that study (Scheme 30) [54].

**Scheme 31:**
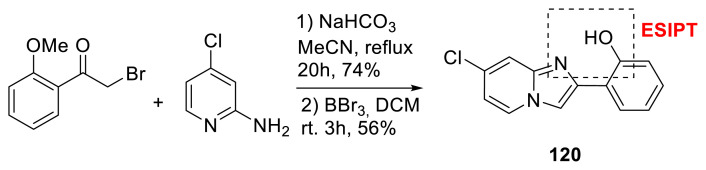
ESIPT-emitted imidazopyridine derivative.

Mutai, Takamizawa, and colleagues developed a 7-chloro-2-(2′-hydroxyphenyl)imidazo[1,2-a]pyridine (**120**) for a superelastochromic crystal. This core may generate light via the ESIPT mechanism, and a similar core was reported by the same research group in 2013 [55]. The solid-state crystallinity of the molecule in the present core was reported by the authors. Due to temperature-independent superelasticity, the suggested molecule displayed reversible chromism, biphasic luminous color changes with emission intensity dependent on domain volume, and stress-sensing. The authors also determined that the features of the molecule separated superelastochromism from other chromisms. They also noted that the advantage of ESIPT molecules is that they have strong emissions with a color that is sensitive to the solid-state molecular configuration (Scheme 31) [56].

## 6. Conclusion: The general ESIPT perspective

Designing an unknown ESIPT structure can be a challenging task, as it requires a thorough understanding of the principles that govern the excited-state intramolecular proton transfer process. However, there are several general strategies that can be used to design ESIPT molecules with desired properties:

1. **Identify suitable donor and acceptor groups:** A hydrogen bond donor and acceptor moieties that are close to one other are required for ESIPT. The donor and acceptor groups might be functional groups like hydroxyl, amino, or carboxyl groups, and they are usually connected by a conjugated pi-system like an aromatic ring or a double bond. The appropriate donor and acceptor groups will be chosen based on the intended emission parameters of the molecule.
2. **Optimize the position of donor and acceptor groups:** The distance between the donor and acceptor groups influences the efficiency of ESIPT emission. As a result, it is critical to optimize the donor and acceptor groups’ positions in order to increase the chance of proton transfer. This is accomplished by changing the length and shape of the conjugated pi-system that connects the donor and acceptor groups.
3. **Incorporate suitable substituents:** By adding appropriate substituents to the aromatic ring or other conjugated system, the emission characteristics of ESIPT molecules may be adjusted. Electron-donating groups such as methoxy or amino groups, for example, can increase the electron density of the pi-system, resulting in red-shifted emission, whereas electron-drawing groups such as nitro or cyano groups can decrease the electron density of the pi-system, resulting in blue-shifted emission.
4. **Test and optimize the molecule:** After designing a prospective ESIPT molecule, it should be produced and examined for emission characteristics. The position and type of the donor and acceptor groups, as well as the substituents on the conjugated pi-system, can be changed to optimize the molecule. This iterative technique may be utilized to create ESIPT molecules with specified emission characteristics that can be employed in a variety of applications.
